# Persistence of SARS-CoV-2–Specific IgG in Children 6 Months After Infection, Australia

**DOI:** 10.3201/eid2708.210965

**Published:** 2021-08

**Authors:** Zheng Quan Toh, Rachel A. Higgins, Lien Anh Ha Do, Karin Rautenbacher, Francesca L. Mordant, Kanta Subbarao, Kate Dohle, Jill Nguyen, Andrew C. Steer, Shidan Tosif, Nigel W. Crawford, Kim Mulholland, Paul V. Licciardi

**Affiliations:** Murdoch Children’s Research Institute, Melbourne, Victoria, Australia (Z.Q. Toh, R.A. Higgins, L.A.H. Do, K. Dohle, J. Nguyen, A.C. Steer, S. Tosif, N.W. Crawford, K. Mulholland, P.V. Licciardi);; The University of Melbourne, Melbourne (Z.Q. Toh, L.A.H. Do, A.C. Steer, S. Tosif, N.W. Crawford, K. Mullholland, P.V. Licciardi); T; he Royal Children’s Hospital, Melbourne (K. Rautenbacher, A.C. Steer, S. Tosif, N.W. Crawford);; World Health Organization Collaborating Centre for Reference and Research on Influenza, The Peter Doherty Institute for Infection and Immunity, Melbourne (F.L. Mordant, K. Subbarao);; London School of Hygiene and Tropical Medicine, London, UK (K. Mulholland)

**Keywords:** SARS-CoV-2, children, IgG, persistence, humoral immune responses, acute respiratory infections, respiratory infections, severe acute respiratory syndrome coronavirus 2, 2019 novel coronavirus disease, coronavirus disease, zoonoses, viruses, coronaviruses, COVID-19, antibodies, Australia

## Abstract

The duration of the humoral immune response in children infected with severe acute respiratory syndrome coronavirus 2 is unknown. We detected specific IgG 6 months after infection in children who were asymptomatic or had mild symptoms of coronavirus disease. These findings will inform vaccination strategies and other prevention measures.

Children <18 years of age account for ≈3% of coronavirus disease (COVID-19) cases worldwide (*1*). Most (70%) children with COVID-19 are asymptomatic or have mild illness; very few require hospitalization (*2*,*3*). The nature and persistence of the immune response generated by children after infection with severe acute respiratory syndrome coronavirus 2 (SARS-CoV-2), the causative agent of COVID-19, is unknown. We investigated the humoral immune response to SARS-CoV-2 in children and adults as part of a longitudinal cohort study in Melbourne, Victoria, Australia.

Nasopharyngeal swab samples of persons with suspected SARS-CoV-2 infection and their close contacts were tested by reverse transcription PCR at The Royal Children’s Hospital in Melbourne during May–October 2020. We invited SARS-CoV-2–positive patients and their household members to participate in this cohort study. We collected blood samples at the time of enrollment, as well as ≈28 days, 3 months, and 6 months later. We obtained written informed consent from parents/guardians and assent from children. The study was conducted with the approval of the Human Research Ethics Committee at The Royal Children’s Hospital (approval no. HREC/63666/RCHM-2019).

To measure IgG, we used a modified 2-step ELISA based on the method described by Amanat et al. (*4*) and the LIAISON SARS-CoV-2 S1/S2 IgG assay (DiaSorin, https://www.diasorin.com). We also conducted a SARS-CoV-2 microneutralization assay on an available subset of samples. For the ELISA, we screened samples using the SARS-CoV-2 receptor-binding domain as the antigen; for potential positive samples, we confirmed results by additional ELISA using S1 antigen. We calculated the results of S1-positive samples according to the World Health Organization SARS-CoV-2 pooled serum standard (standard provided by the National Institute for Biological Standards and Control, South Mimms, UK) and reported data as ELISA units per milliliter. We set a seropositivity cutoff at 1.5 ELISA units/mL on the basis of results of archived serum samples taken before the pandemic. We then conducted the LIAISON assay according to the manufacturer’s instructions and the microneutralization assay as described by Tosif et al. (*5*) (Appendix). 

During May 10, 2020–October 28, 2020, we recruited a cohort of 134 children (0–18 years of age) and 160 adults (19–73 years of age). We included only participants with a positive PCR result for SARS-CoV-2 or who were seropositive at the first timepoint (median 11 days after diagnosis, range 5–13 days) and had blood samples for >2 timepoints. At the first timepoint, 4 adults had negative PCR results but positive serologic results; of these adults, 3 had borderline seropositive antibody levels.

By February 2021, we had identified 54 SARS-CoV-2–positive participants: 22 children (median age of 4 years, range 0–18 years) and 32 adults (median age of 37 years, range 22–73 years). In total, 5 (23%) children and 2 (6%) adults were asymptomatic; the rest had mild symptoms, and none were hospitalized. The median duration of follow-up after diagnosis was 195 days (range 188–213 days) for children and 194 days (range 183–212 days) for adults.

By day 43 (range 27–79), 15/19 (79%) children and 26/28 (93%) adults had seroconverted. These participants remained seropositive for >90 days (Figure, panels A, B). By day 195 (≈6 months), 14/17 (82%) of children and 18/21 (86%) of adults were seropositive; however, from day 43 to 195, geometric mean antibody concentration decreased ≈2-fold in both groups (Figure, panel C). We observed no significant differences in geometric mean antibody concentration from day 43 (range 27–79) to day 194 (range 183–212), nor from 93 (range 27–79) to day 194 (range 183–212), for either children or adults (Figure, panels A, B). The seropositivity and antibody levels were also not significantly different between children and adults at all timepoints (Figure, panel C; Appendix Figure 1). Seropositive samples defined by our in-house ELISA correlated with results from the LIAISON assay and neutralizing antibody assay (Appendix Figures 2, 3). In total, 4/19 (21%) children and 2/28 (7%) adults did not seroconvert; however, we could not rule out other SARS-CoV-2–related immune responses, such cellular or mucosal mechanisms (*5*,*6*).

**Figure:**
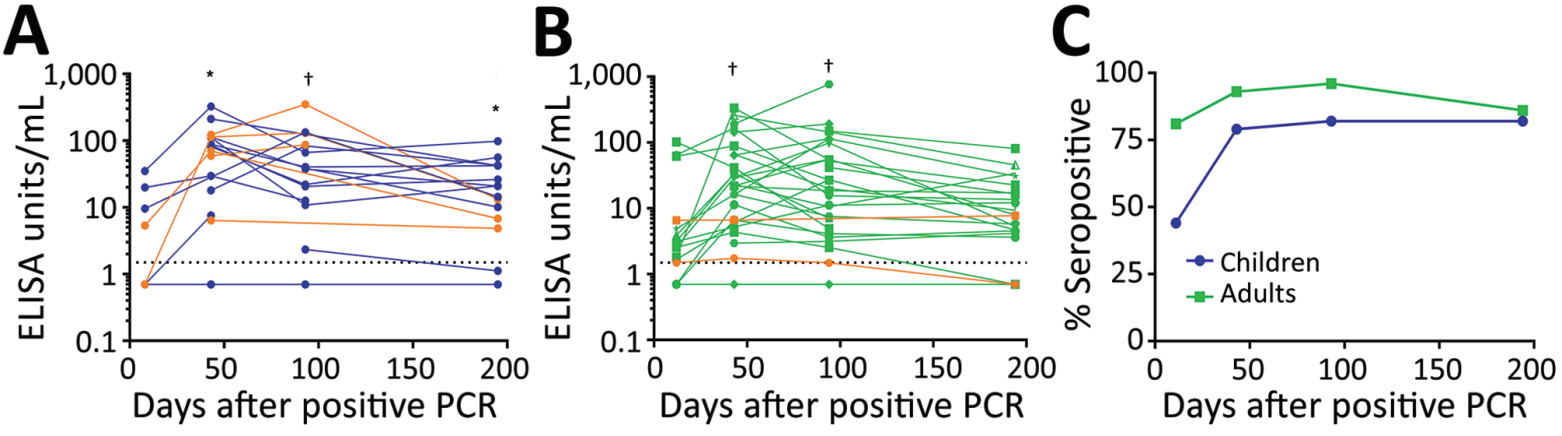
Persistence of IgG responses against severe acute respiratory syndrome coronavirus 2 in children and adults, Australia, 2020–2021. Patients tested positive by PCR, ELISA, or both. A) Antibody responses of 22 children 0–18 years of age. B) Antibody responses of 32 adults 22–73 years of age. Orange points and lines indicate asymptomatic cases; blue points and lines indicate symptomatic cases in children; green points and lines indicate symptomatic cases in adults. Dotted lines indicate seropositivity cutoff. C) Seropositivity rates in 22 children and 32 adults. Blue points and lines indicate all children, regardless of symptoms; green points and lines indicate all adults, regardless of symptoms. *p<0.05; †p<0.01 (compared with the fist timepoint [day 11]).

We found that, similar to the adults in this cohort and those in previous studies (7,8), SARS-CoV-2–positive children with no or mild symptoms mounted strong and durable humoral responses that persisted for >6 months. Our study was limited by the relatively small sample size; in addition, only a subset of samples was available for the microneutralization assay. In conclusion, our data indicate that SARS-CoV-2–positive children have a persistent antibody response for >6 months. The roles and durations of other components of the immune system (such as the cellular and mucosal responses) during SARS-CoV-2 infection remain undetermined. These results will inform vaccination strategies and other public health measures. 

Appendix Additional information on the persistence of SARS-CoV-2–specific IgG in children 6 months after infection, Australia.
