# Supplementary material for: Persistence of SARS-CoV-2–Specific IgG in Children 6 Months After Infection, Australia
Source: Emerg Infect Dis. 2021 Aug;27(8):2233–5. doi: 10.3201/eid2708.210965 (PMC8314814; doi:10.3201/eid2708.210965)
Supplement: Appendix — Additional information on the persistence of SARS-CoV-2–specific IgG in children 6 months after infection, Australia. [file 21-0965-Techapp-s1.pdf]

Article DOI: https://doi.org/10.3201/eid2708.210965

Persistence of SARS-CoV-2–Specific IgG in Children 6 Months After Infection, Australia

Appendix

Appendix Methods

ELISA

We used a modified 2-step ELISA based on the method described by Amanat et al. (*4*) but used S1 instead of the trimeric spike protein to determine the antibody response against SARS-CoV-2. This assay has 100% specificity and 93% sensitivity for SARS-CoV-2 antibodies and was granted Emergency Use Authorization from the US Food and Drug Administration (Reference 1 in Appendix). Briefly, 96-well high binding plates (Thermo Fisher Scientific, https://www.thermofisher.com) were coated with SARS-CoV-2 receptor-binding domain (RBD) or S1 (Sino Biological Inc., https://www.sinobiological.com) diluted in phosphate-buffered saline (PBS) at 2 μg/mL and then incubated at 4°C overnight. The next day, plates were washed with PBS containing 0.1% (v/v) Tween20 (PBS-T)(MP Biomedicals, https://www.mpbio.com) and blocked with PBS containing 0.1% Tween and 10% (w/v) skim milk (PBS-TSM) for 1 h at room temperature (RT). Serum samples prepared in PBS-TSM were first screened at 1:50 dilution in duplicates, and potential seropositive samples were then confirmed with either RBD or S1 titration assay starting at 1:80 with 4-fold serial dilutions. The blocking solution was removed and 50 μl of each serial dilution was added to the plates for 2 h at RT. The plates were then washed 3 times with 200 μl per well of PBS-T. Goat anti-human IgG horseradish peroxidase conjugated secondary antibody (Southern Biotech, https://www.southernbiotech.com) was prepared in PBS-TSM (1:10,000), and 50 μl of this secondary antibody was added to each well for 1 h. Plates were washed with PBS-T followed by distilled water and 50 μL of 3.3′, 5.5′-tetramethylbenzidine (SeraCare, https://www.seracare.com) substrate solution was added for 9 min. The reaction was stopped by the addition of 50 μL of 1M phosphoric acid (Sigma Aldrich, https://www.sigmaaldrich.com) and optical densities measured using a microplate reader (BioTek Instruments, Inc., https://www.biotek.com) at 450 nm (630 nm reference filter).

Seropositive samples were titrated and calculated based on a World Health Organization SARS-CoV-2 pooled serum standard obtained from the National Institute of Biologic Standards and Controls, United Kingdom. Samples with optical density readings (at 450 nm) that exceeded a cutoff of 0.5 units based on the RBD screening assay (based on 40 pre-pandemic sera) were considered to be potentially positive and were subjected to sample titration using S1 protein. Seropositive cutoff for the confirmatory assay was set at 1.5 ELISA Units/mL. Seronegative samples from the screening assay were assigned half of the seropositive cutoff value.

Liaison SARS-CoV-2 S1/S2 IgG Assay

The quantitative commercial assay for the detection of IgG antibodies against S1/S2 antigens of SARS-CoV-2 was performed according to the manufacturer instructions (LIAISON SARS-CoV-2 S1/S2 IgG assay; DiaSorin, https://www.diasorin.com). Data was reported as Assay Units/mL. Seronegative samples by Diasorin were assigned half the cutoff value (<12 AU/mL).

SARS-CoV-2 Microneutralisation Assay

SARS-CoV-2 isolate CoV/Australia/VIC01/202027 passaged in Vero cells was stored at −80°C. Serial 2-fold dilutions of heat-inactivated plasma were incubated with 100 50% tissue culture infectious dose of SARS-CoV-2 for 1 h and residual virus infectivity was assessed in quadruplicate wells of Vero cells; viral cytopathic effect was read on day 5. The neutralizing antibody titer is calculated using the Reed/Muench method (Reference 2 in Appendix).

Statistical Analysis

The S1-specific IgG antibody levels between children and adults, as well as between each timepoint within children or adult were compared using Mann-Whitney U test. For correlation analysis, antibody titers and concentrations were log-transformed and analyzed using Pearson’s correlation analysis. Fisher exact test was used to compare the seropositivity rate. All analyses were performed with GraphPad Prism version 7.0 (GraphPad Software, https://www.graphpad.com). A $p<0.05$ was considered significant.

References

1. Icahn School of Medicine at Mount Sinai. Mount Sinai’s blood test to detect antibodies to COVID-19 receives emergency use authorization from U.S. Food and Drug Administration. 2020 [cited 2021 Apr 27]. https://www.mountsinai.org/about/newsroom/2020/mount-sinais-blood-

test-to-detect-antibodies-to-covid19-receives-emergency-use-authorization-from-us-food-and-drug-administration-pr.

2. Houser KV, Gretebeck L, Ying T, Wang Y, Vogel L, Lamirande EW, et al. Prophylaxis with a Middle East Respiratory Syndrome Coronavirus (MERS-CoV)-specific human monoclonal antibody protects rabbits from MERS-CoV infection. J Infect Dis. 2016;213:1557–61. PubMed https://doi.org/10.1093/infdis/jiw080

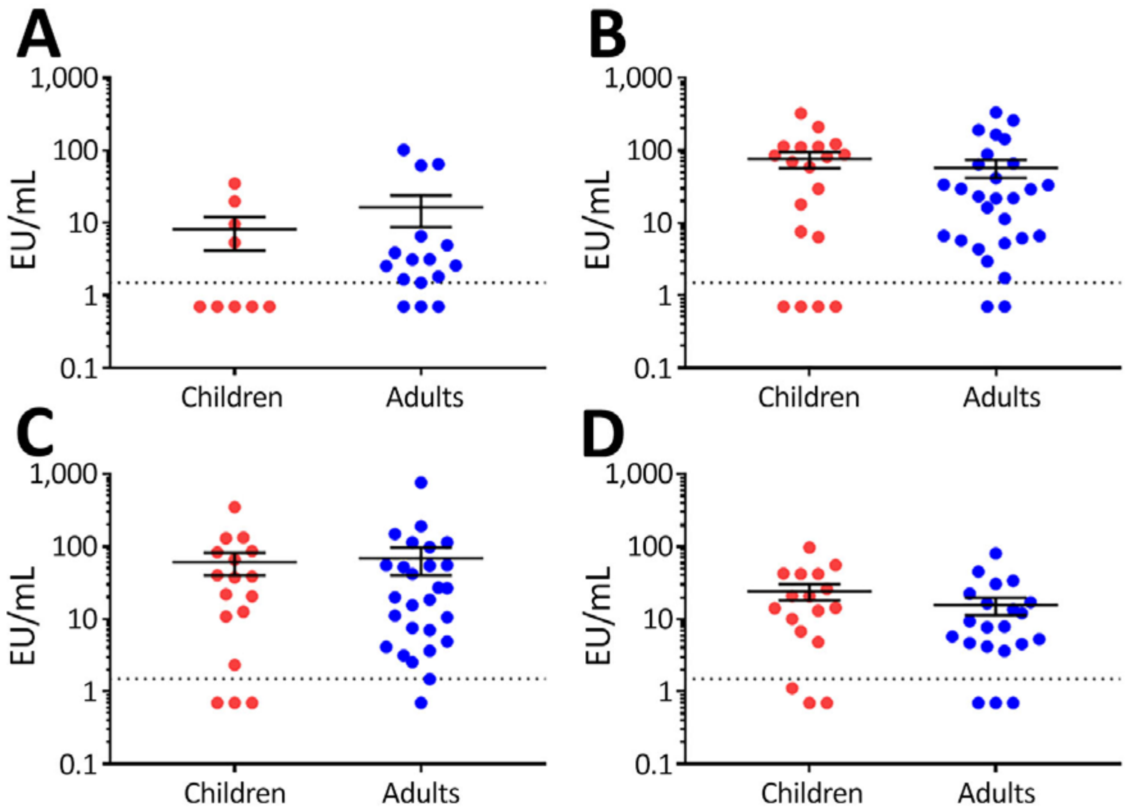

Appendix Figure 1. Comparison of IgG responses against severe acute respiratory syndrome coronavirus 2 between children and adults at each timepoint, Australia, 2020–2021. Dotted lines indicate seropositivity cutoff. EU, ELISA units.

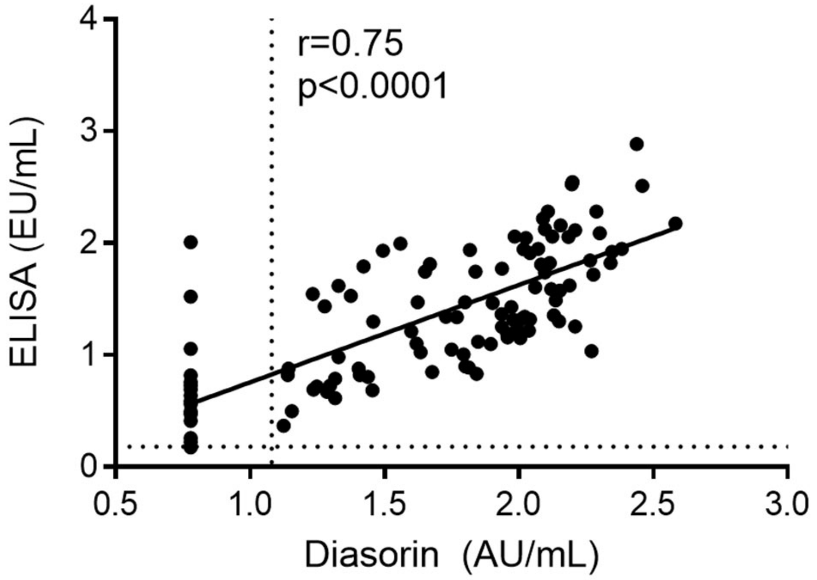

Appendix Figure 2. Correlation between log-transformed results of 2-step ELISA and LIAISON SARS-CoV-2 S1/S2 IgG assay (DiaSorin, https://www.diasorin.com) using Pearson's correlation analysis, Australia, 2020–2021. Data shown for 113 samples from 53 persons. Horizontal dotted lines indicate seropositivity cutoffs for Diasorin; vertical dotted lines indicate seropositivity cutoffs for ELISA. AU, assay units; EU, ELISA units.

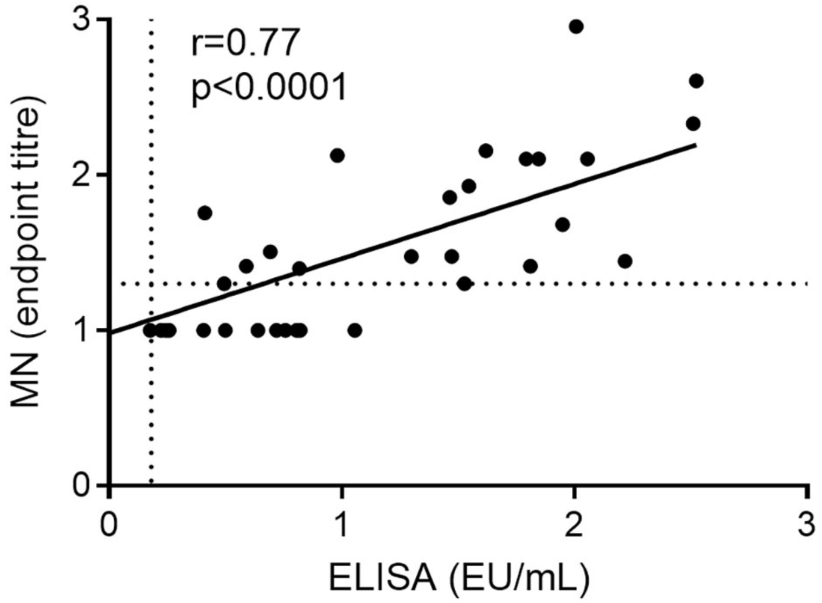

Appendix Figure 3. Correlation between log-transformed results of in-house ELISA and microneutralization assay for severe acute respiratory syndrome coronavirus 2 using Pearson's correlation analysis, Australia, 2020–2021. Paired data shown for 47 samples. Horizontal dotted lines indicate seropositivity cutoffs for neutralization assay; vertical dotted lines indicate seropositivity cutoffs for ELISA. EU, ELISA units; MN, microneutralization.
